# Supplementary material for: SCAPTURE: a deep learning-embedded pipeline that captures polyadenylation information from 3′ tag-based RNA-seq of single cells
Source: Genome Biol. 2021 Aug 10;22:221. doi: 10.1186/s13059-021-02437-5 (PMC8353616; doi:10.1186/s13059-021-02437-5)
Supplement: Supplementary file 1 — Additional file 1: Figures S1-S10 with figure legends. [file 13059_2021_2437_MOESM1_ESM.docx]

**
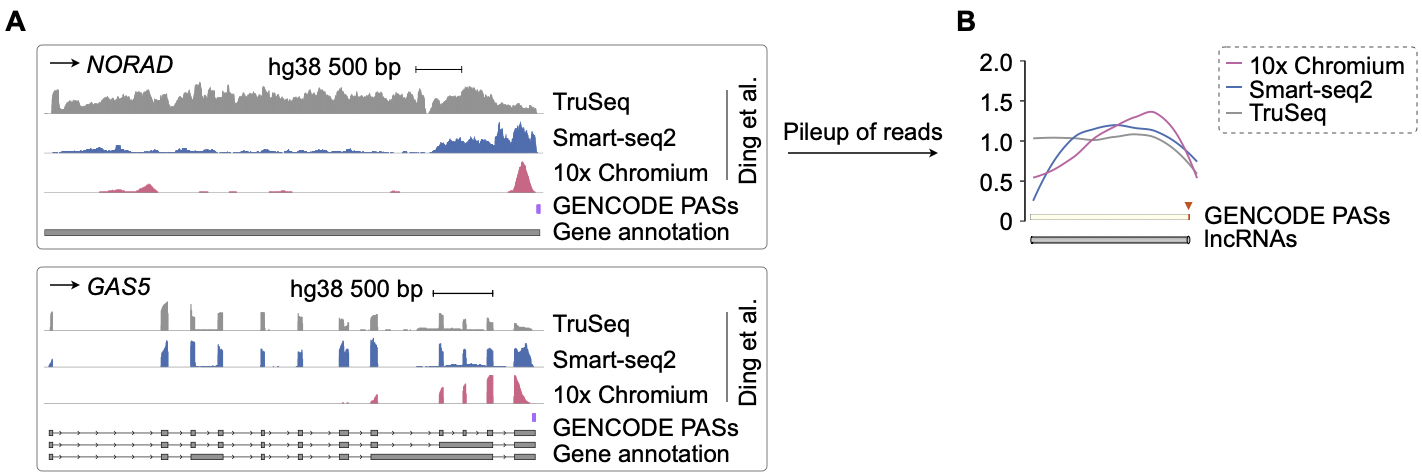
Fig. S1. A 3'-biased distribution of 10x Chromium scRNA-seq on long noncoding RNA (lncRNA) genes.**

(A) Comparison of human PBMC transcriptome profiling on lncRNA genes with different deep sequencing datasets. Wiggle tracks showed an enrichment of 10x Chromium reads (rose) at the 3' ends of the *NORAD* and *GAS5* gene loci, close to known GENCODE PASs, while reads of full-length TruSeq RNA-seq (gray) and Smart-seq2 (dark blue) were covered the whole gene bodies. Data were retrieved from published PBMC TruSeq RNA-seq, Smart-seq2 or 10x Chromium scRNA-seq.

(B) Distribution of deep sequencing reads on lncRNA genes. Pileup of deep sequencing reads from the same published datasets also indicated the characteristic enrichment of 10x Chromium reads (rose) at 3' ends of lncRNA genes, compared to the whole coverage of lncRNA gene bodies of TruSeq RNA-seq (gray) and Smart-seq2 (dark blue). The distribution of PASs was calculated from GENCODE.

**
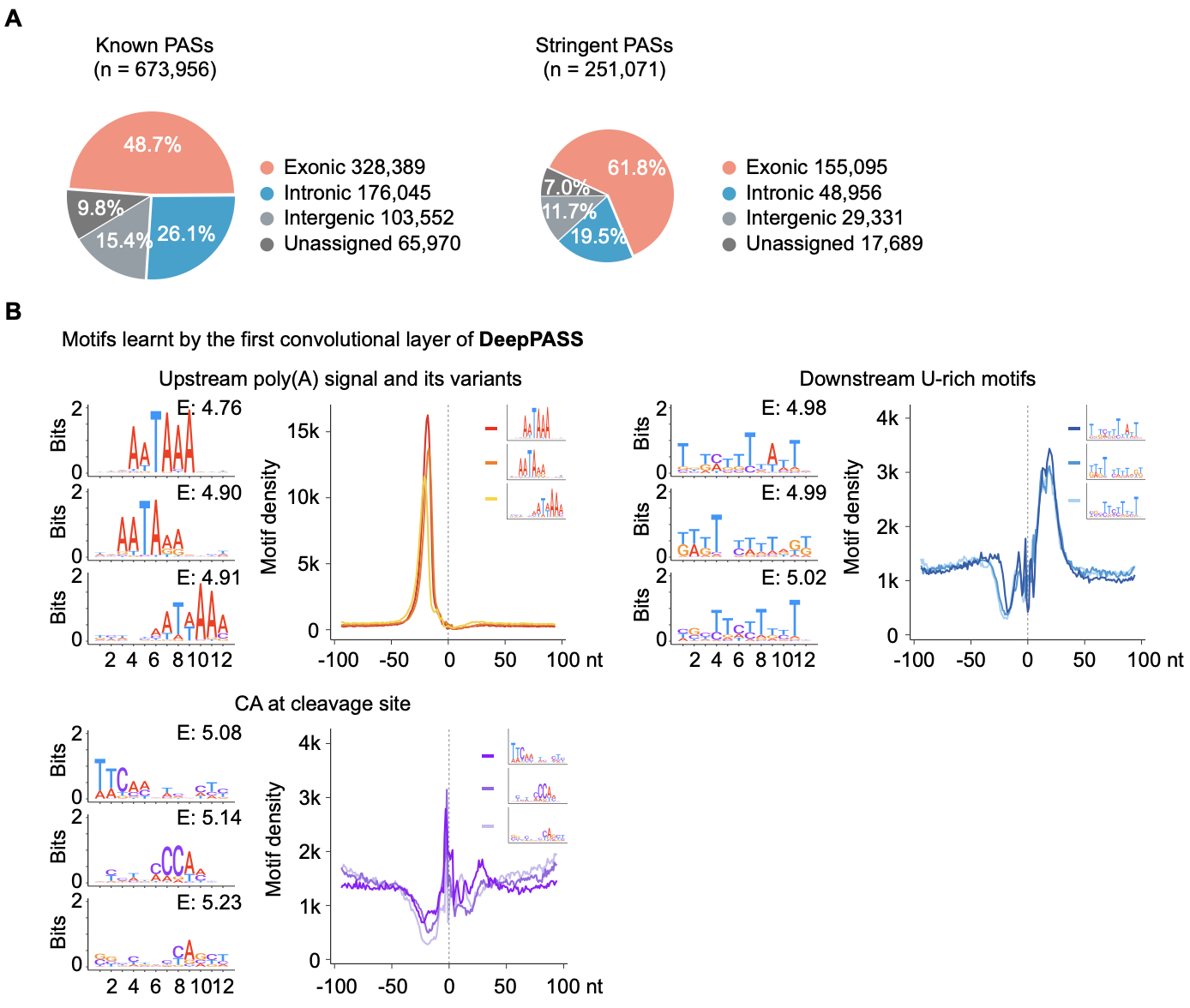
Fig. S2. Poly(A) site training data collection and learnt poly(A) signals of DeepPASS model.**

(A) Distribution of PASs in the human genome (GENCODE v34 hg38). Left, distribution of known PASs. Right, distribution of stringent PASs.

(B) Active motifs of DeepPASS model. Three featured PAS motifs, including upstream poly(A) signal AAUAAA and its variants, downstream U-rich motifs and CA nucleotides at cleavage sites were all identified with DeepPASS model. Motif distribution was calculated on 200-bp sequences around stringent PASs.

**
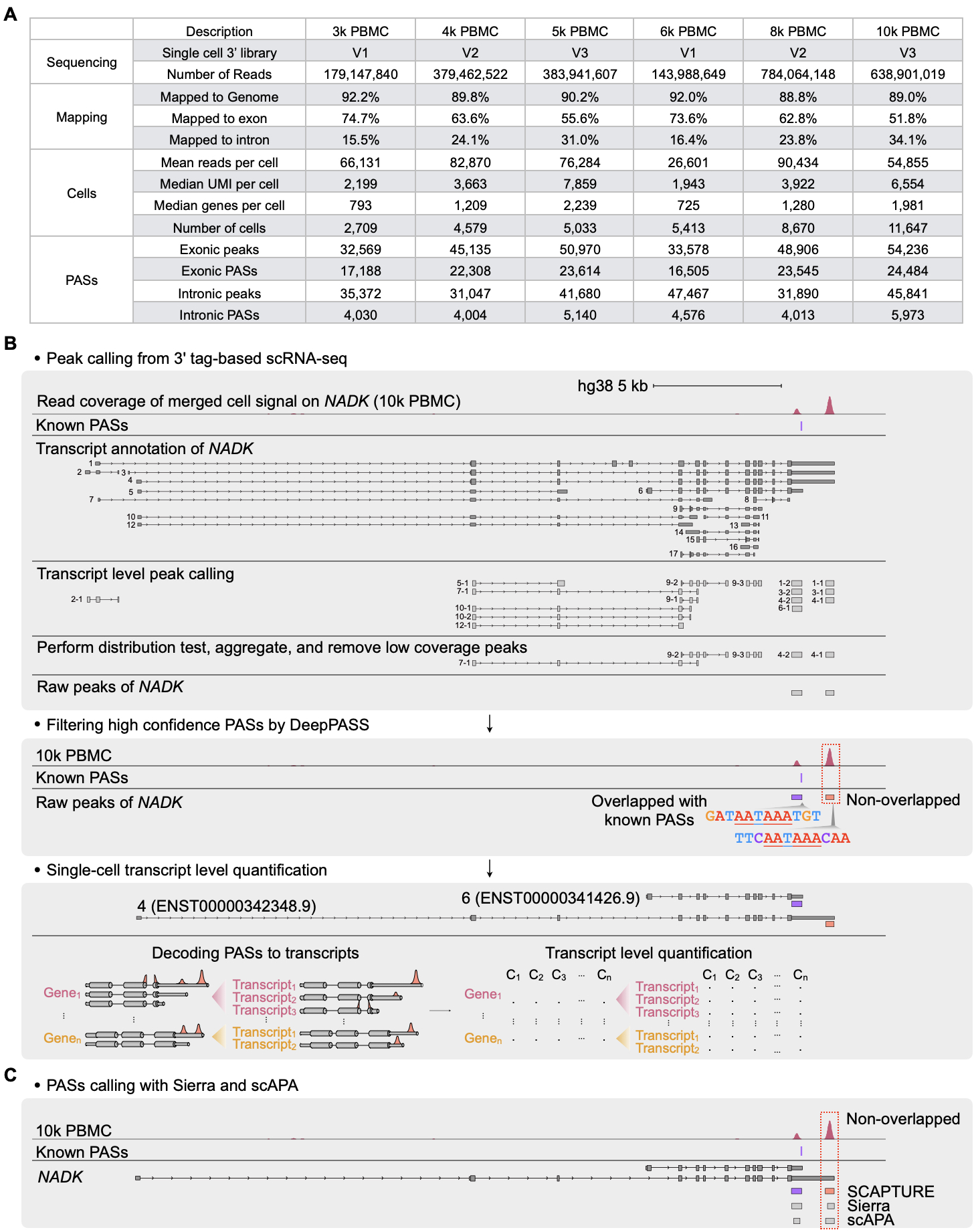
Fig. S3.** **Application of SCAPTURE to call PASs in** **six PBMC scRNA-seq datasets from 10x Genomics.**

(A) Summary of six PBMC scRNA-seq datasets from 10x Genomics. Information of sequencing, mapping statistics, cell identification, and peaks/PASs called by SCAPTURE from all six samples, 3k, 4k, 5k, 6k, 8k and 10k, were individually listed.

(B) A step-by-step analysis by SCAPTURE to call PASs in the *NADK* gene locus. Results from the 10k PBMC dataset were illustrated. Top, peak calling. Read of 10k PBMC dataset from 10x Genomics was applied to call peaks according to transcript annotation of the *NADK* gene. For simplicity, each transcript was marked with a number, and each peak called from specific transcript was further indexed according to the transcript number. For example, the peak labeled with “4-1” was the first peak in transcript labeled with “4”. Peaks were further tested and aggregated for subsequent analysis. Middle, filtering high-confidence PASs by DeepPASS. Two peaks from the peak calling step were both passed by DeepPASS model prediction, and further compared with known PASs (Fig. 2A; Additional file 1: Fig. S2A). Bottom, PAS-based transcript quantification. Different APA transcripts can be determined by distinct PASs, and thus quantified PASs by SCAPTURE could be used to represent differential transcript expression (DTE).

(C) Similar PAS calling by SCAPTURE, Sierra and scAPA in the *NADK* locus.

**
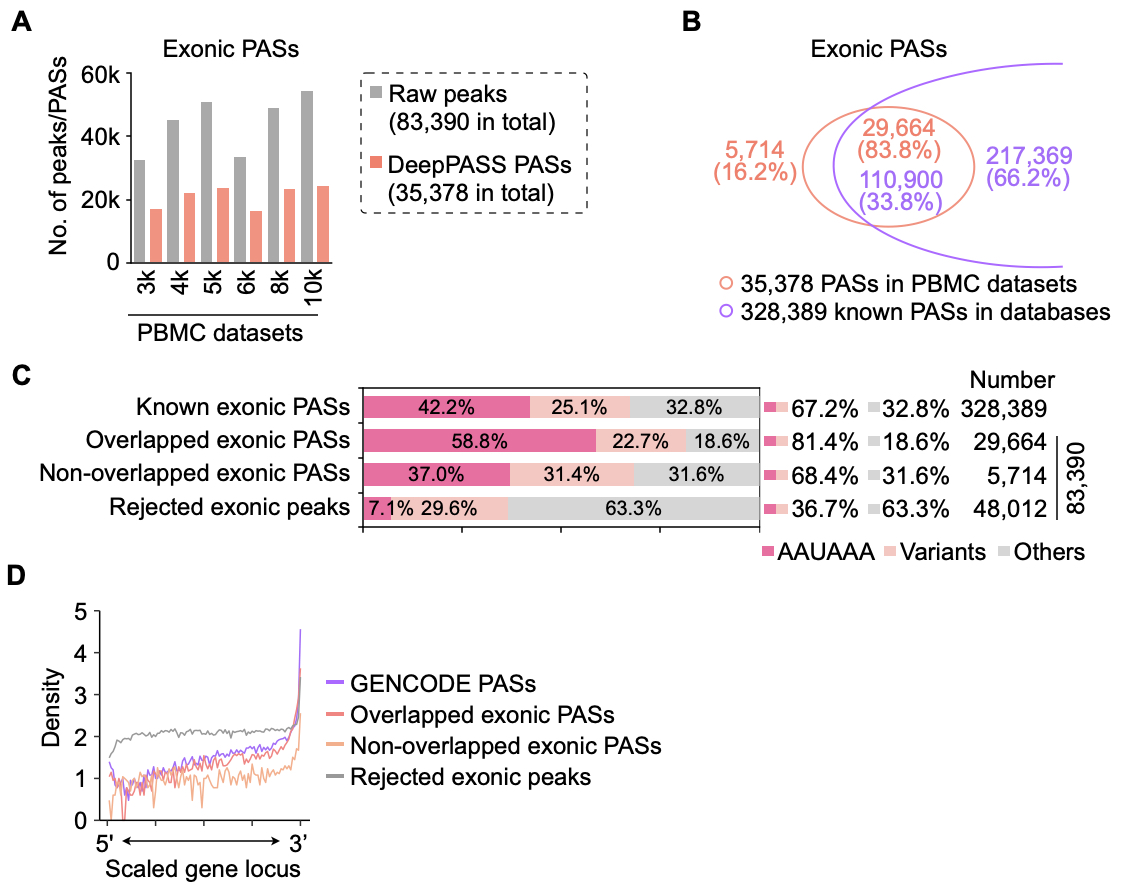
Fig. S4. Feature analyses of SCAPTURE-identified exonic PASs.**

(A) Statistics of exonic peak calling and PAS filtering individually in six PBMC datasets (10x Genomics).

(B) Overlapping of SCAPTURE-identified exonic PASs combined from six PBMC datasets (10x Genomics) with known exonic PASs.

(C) Frequencies of canonical AAUAAA motif and its variants in known, overlapped, non-overlapped and rejected exonic PASs/peaks.

(D) Distribution of known, overlapped, non-overlapped and rejected exonic PASs/peaks along gene body.

**
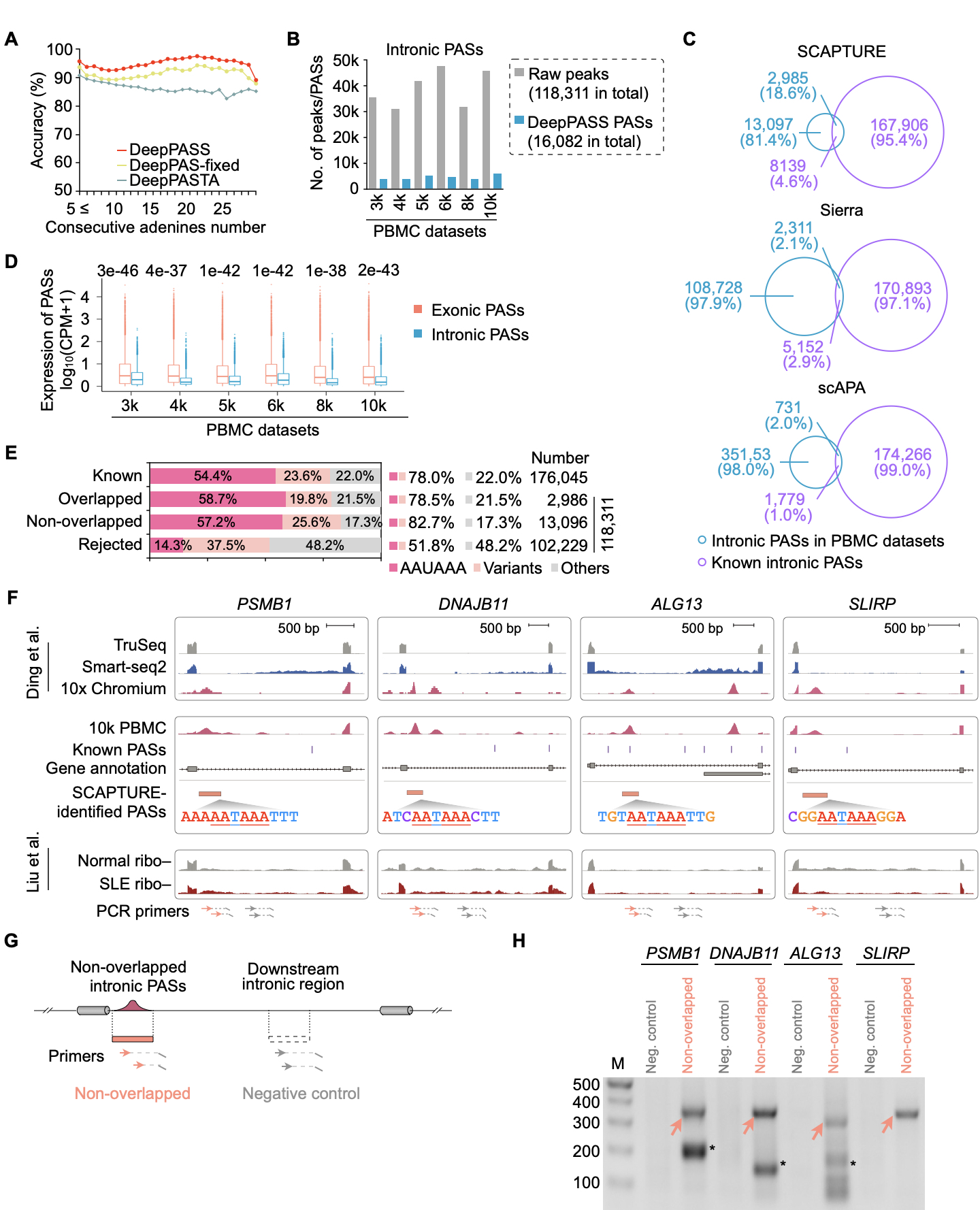
Fig. S5. Evaluation of SCAPTURE-identified intronic PASs.**

(A) Efficiency of DeepPASS identifying internal priming sequences. True negative rates of different models in predicting PASs from generated pseudo internal priming sites.

(B) Statistics of intronic peak calling and PAS filtering individually in six PBMC datasets (10x Genomics).

(C) Overlapping of intronic PASs combined from six PBMC datasets (10x Genomics) with known intronic PASs. Top, SCAPTURE-identified intronic PASs. Middle, Sierra-identified intronic PASs. Bottom, scAPA-identified intronic PASs.

(D) Comparison of quantified exonic and intronic PASs identified with SCAPTURE individually in six PBMC datasets (10x Genomics). Statistical significance was assessed with two-tailed Welch Two Sample *t*-test. Boxplots were shown as median and interquartile range (IQR).

(E) Frequencies of canonical AAUAAA motif and its variants in known, overlapped, non-overlapped and rejected intronic PASs/peaks.

(F) Validation of SCAPTURE-identified non-overlapped intronic PASs. Four such intronic PASs (salmon rectangle) in the *PSBM1*, *DNAJB11*, *ALG13* and *SLIRP* gene loci were highlighted with their polyadenylation signals (AAUAAA motif). Top panel, TruSeq RNA-seq (gray), Smart-seq2 (dark blue) and 10x Chromium (rose) scRNA-seq from published datasets [17]. Middle panel, wiggle tracks from one of 10x Genomics PBMC scRNA-seq datasets (the 10k PBMC dataset) were indicated. Bottom panel, Ribo– RNA-seq datasets from normal (gray) and SLE patient (dark red) PBMC cDNA samples [25]. PCR primers were shown in the bottom for experimental validation.

(G) Schematic diagram of primer design for validation of SCAPTURE-identified non-overlapped intronic PASs.

(H) Corresponding PCR products of four non-overlapped intronic PASs (salmon arrow) were shown with correct sizes. As negative controls, no PCR products from downstream regions of these SCAPTURE-identified non-overlapped intronic PASs were detected. Mixed PBMC RNA samples from SLE patients previously examined by Liu et al [25] were used for this validation. Of note, additional nested primers were designed to validate all these PASs. Asterisks, non-specific PCR bands.

**
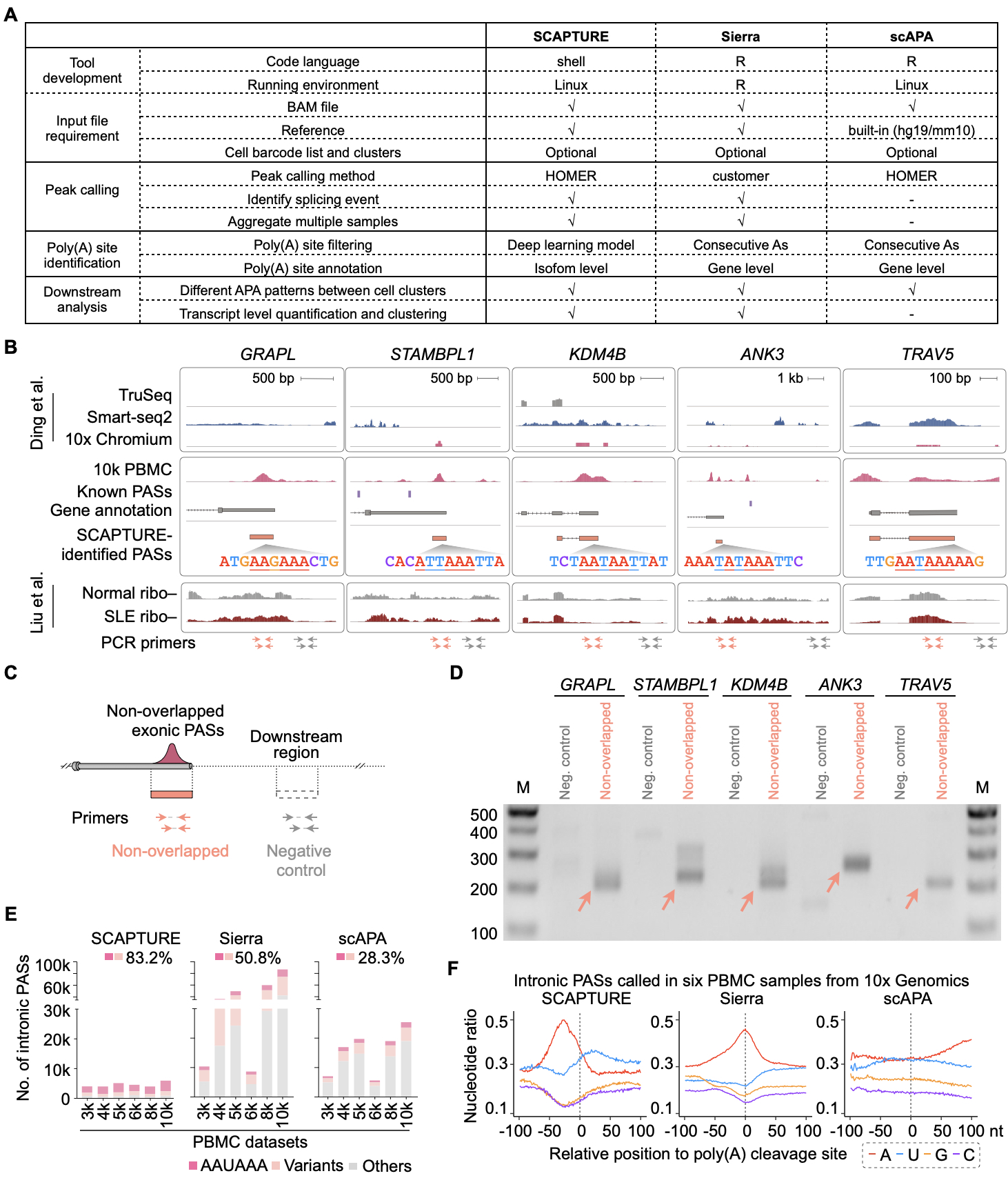
Fig. S6.** **Comparison of SCAPTURE with two other PAS-detecting methods.**

(A) Comparison of parameters in SCAPTURE with Sierra and scAPA methods. Sierra and scAPA were recently-reported to identify PASs from scRNA-seq datasets.

(B) Examples of non-overlapped exonic PASs uniquely-identified by SCAPTURE. Five such cases (salmon rectangle) in the *GRAPL*, *STAMBPL1*, *KDM4B*, *ANK3*, and *TRAV5* gene loci were highlighted with their polyadenylation variant signals. Top panel, TruSeq RNA-seq (gray), Smart-seq2 (dark blue) and 10x Chromium (rose) scRNA-seq from published datasets [17]. Middle panel, wiggle tracks from one of 10x Genomics PBMC scRNA-seq datasets (the 10k PBMC dataset) were indicated. Bottom panel, Ribo– RNA-seq datasets from normal (gray) and SLE patient (dark red) PBMC cDNA samples [25]. PCR primers were shown in the bottom for experimental validation.

(C) Schematic diagram of primer design for validation of non-overlapped exonic PASs uniquely identified by SCAPTURE.

(D) Corresponding PCR products of five non-overlapped exonic PASs uniquely identified by SCAPTURE were shown with correct sizes (salmon arrow). As negative controls, no PCR products from downstream regions of these PASs were detected. Mixed PBMC RNA samples from SLE patients previously examined by Liu et al [25] were used for this validation. Of note, additional nested primers were designed to validate all these PASs.

(E) Comparison of signature poly(A) signal motifs of intronic PASs identified by SCAPTURE, Sierra or scAPA from six PBMC datasets (10x Genomics).

(F) Nucleotide distribution of sequences around intronic PASs identified by SCAPTURE, Sierra and scAPA from six PBMC datasets (10x Genomics). Upstream (–) 100 bp to downstream (+) 100 bp sequences of PASs were analyzed.

**
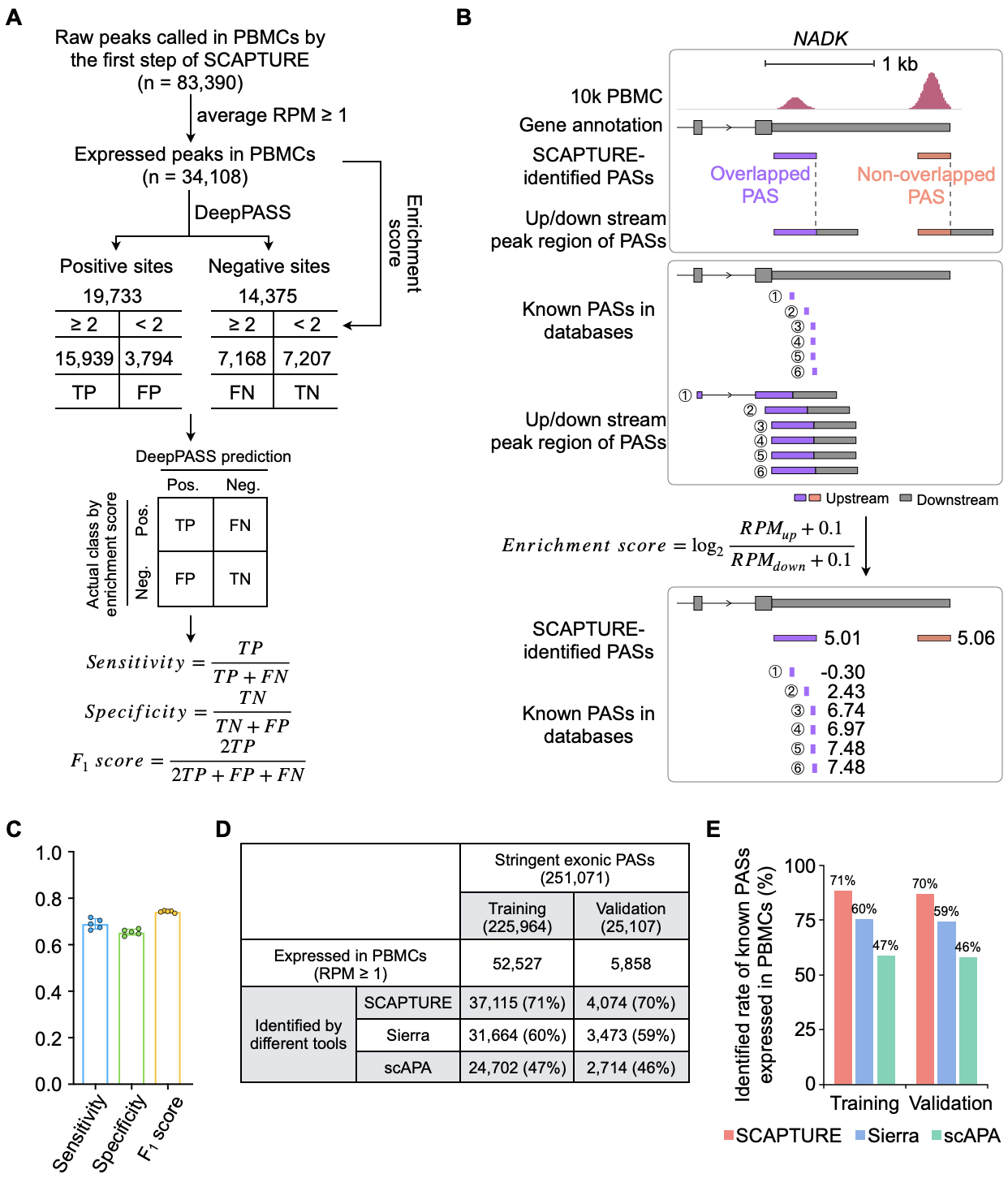
Fig. S7. Calculation of DeepPASS’s sensitivity and specificity.**

(A) Pipeline of using enrichment scores to evaluate sensitivity and specificity of DeepPASS.

(B) Schematic diagram of enrichment score calculation. One (purple) of two SCAPTURE-identified PASs was overlapped with six known PASs at the last exon of *NAKD*, and the other one (salmon) is not overlapped with known PAS annotation.

(C) Sensitivity, specificity and F_1_ scores of DeepPASS calculated individually from PBMC scRNA-seq datasets (10x Genomics). Data points showed results calculated with different enrichment score cutoffs.

(D) Statistics of identified PASs from training and validation sets (split with a 9:1 ratio from stringent PASs) by SCAPTURE, Sierra and scAPA.

(E) Bar plots of identified PAS rates by SCAPTURE, Sierra and scAPA (statistics from D).

**
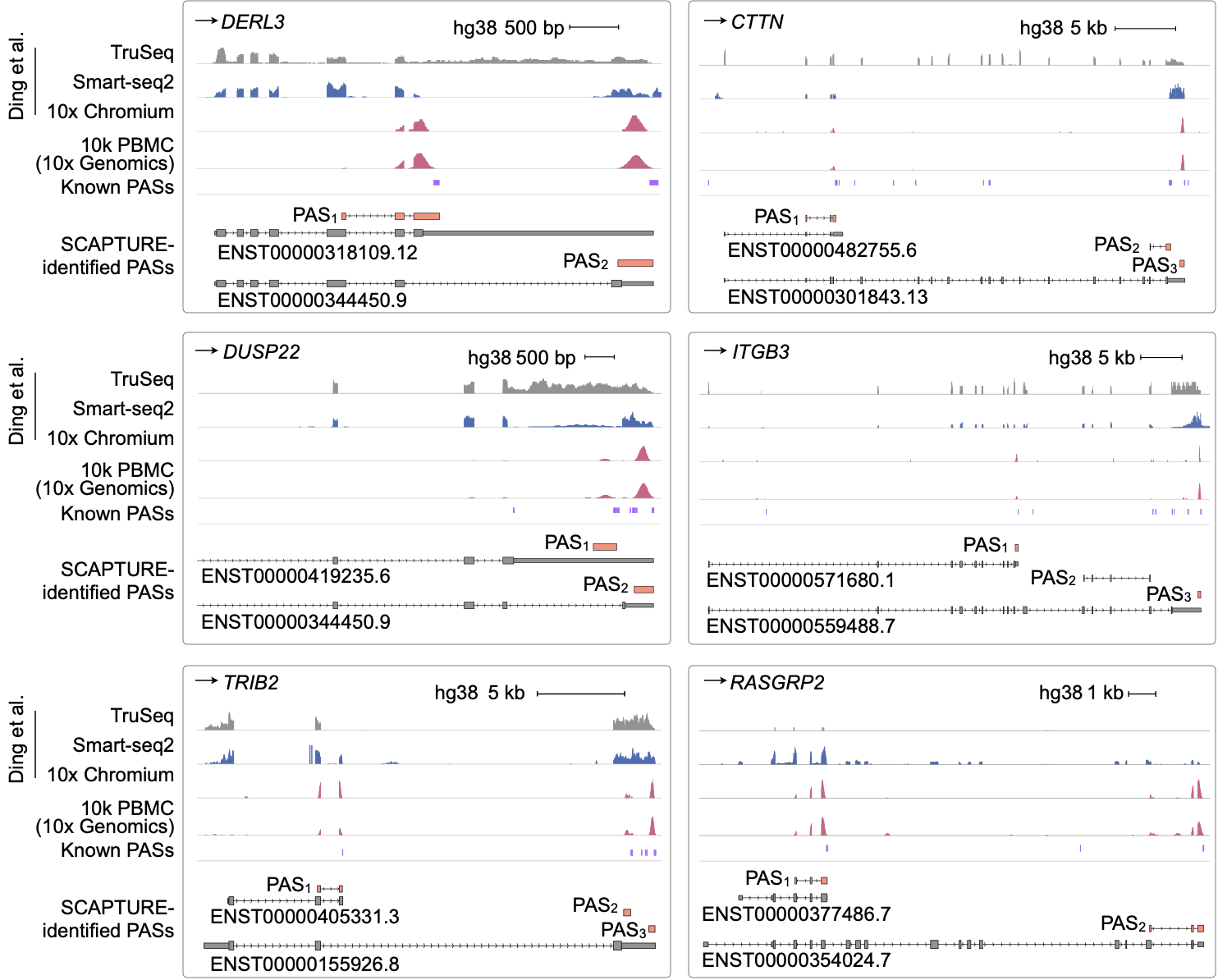
Fig. S8. Principle of using quantified PASs to represent APA transcript expression.**

Multiple PASs could be determined by SCAPTURE at examined gene loci to indicate different transcript expression (DTE) with distinct PAS usage. In this case, values of quantified PASs could be used to represent APA transcript expression. Data were retrieved from published PBMC TruSeq RNA-seq (gray), Smart-seq2 (dark blue) or 10x Chromium (rose, middle) scRNA-seq, and the 10k PBMC dataset from 10x Genomics (rose, bottom). Transcript annotation was from GENCODE (v34 hg38).

**
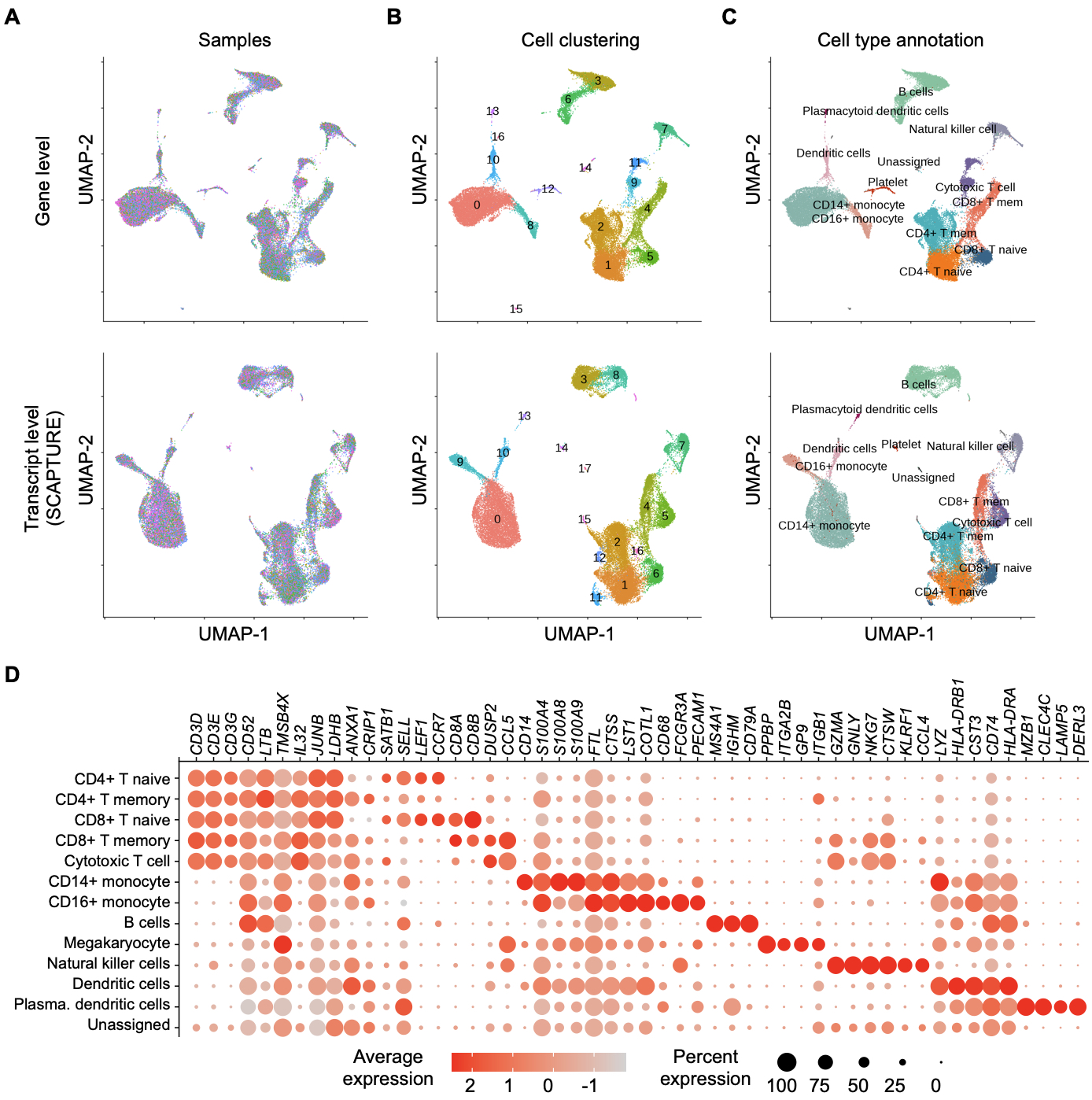
Fig. S9. Parallel comparison of single-cell analysis results with developed SCAPTURE method and canonical Seurat method.**

(A) UMAP plots to show integration of different PBMCs samples with features extracted from DTE by SCAPTURE (bottom) or those from DGE by Seurat (top).

(B) UMAP plots to show unsupervised cell clustering with DTE by SCAPTURE (bottom) or with DGE by Seurat (top).

(C) UMAP plots to show unsupervised cell type clustering with annotation of each type of cell cluster with DTE by SCAPTURE (bottom) or with DGE by Seurat (top).

(D) Comparison of marker gene expression among different cell types.

**
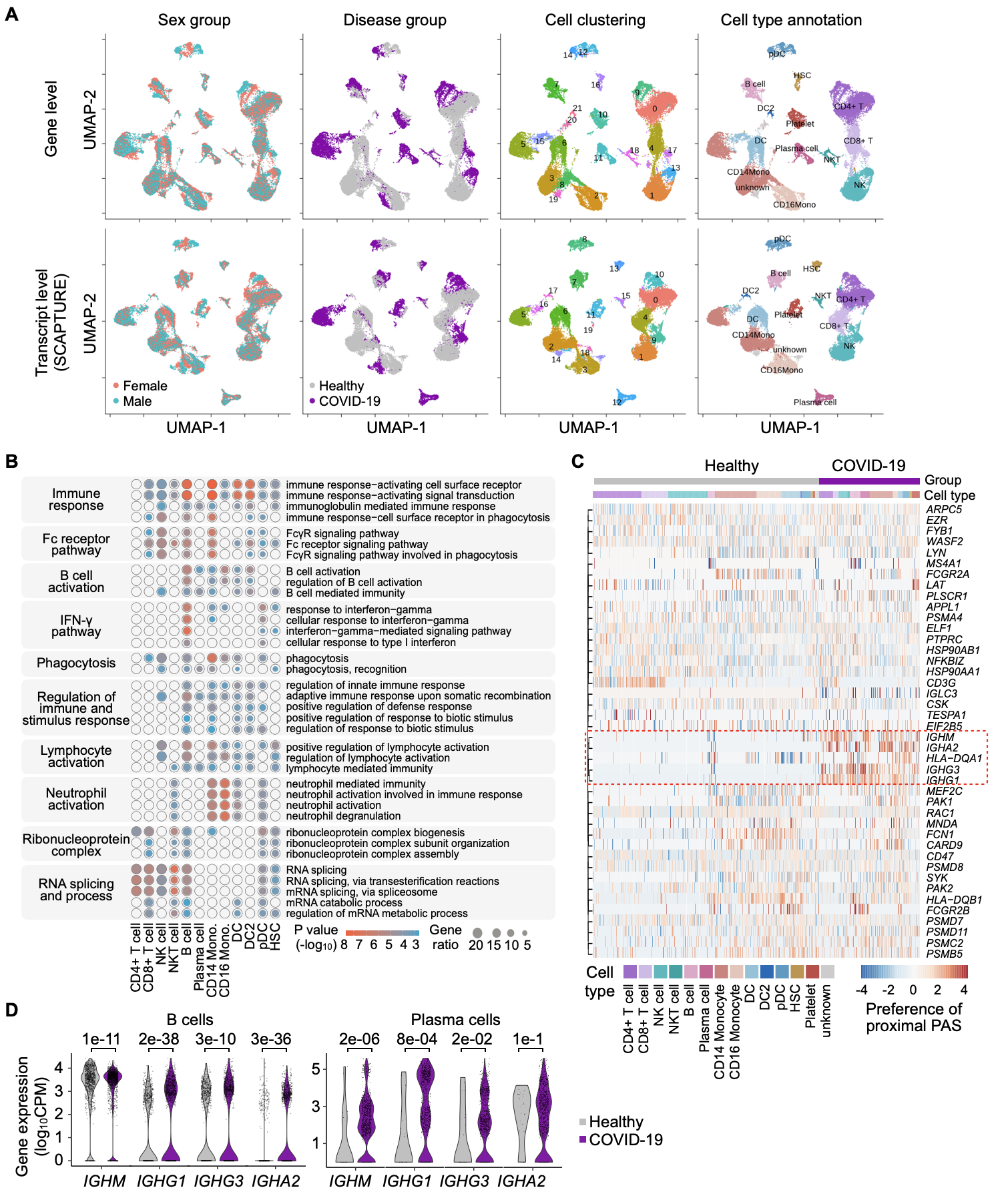
Fig. S10. Analyses of PBMC scRNA-seq datasets of healthy individuals and COVID-19 patients.**

(A) UMAP plots to show integration of PBMC scRNA-seq datasets from healthy individuals and COVID-19 patients by DGE (top panel) and DTE (bottom panel). Single cells were labeled by sex group, disease group, unsupervised clustering and annotated cell types from left to right, respectively.

(B) GO analysis of gene sets with altered PAS usage between healthy individuals and COVID-19 patients in each type of PBMC immune cells.

(C) Profiling of immune response-related genes (gene set from B) of single cells in PBMCs scRNA-seq datasets from healthy individuals and COVID-19 patients. A preferential proximal PAS usage of some immunoglobulin genes in COVID-19 patients was highlighted with dashed line in red.

(D) Gene expression of immunoglobulin genes in B cells (left) and plasma cells (right) from healthy individuals and COVID-19 patients. Statistical significance was assessed with Seurat (“methods” section).
